# Calpain in Traumatic Brain Injury: From Cinderella to Central Player

**DOI:** 10.3390/cells14161253

**Published:** 2025-08-14

**Authors:** Carla Schallerer, Stephan Neuschmid, Barbara E. Ehrlich, Declan McGuone

**Affiliations:** 1 School of Medicine and Health, Technical University of Munich, 81675 Munich, Germany; carla.schallerer@yale.edu (C.S.); stephan.neuschmid@yale.edu (S.N.); 2 Department of Pharmacology, Yale School of Medicine, New Haven, CT 06510, USA; 3 Department of Pathology, Yale School of Medicine, New Haven, CT 06510, USA

**Keywords:** Traumatic Brain Injury, calcium dysregulation, excitotoxicity, axon degeneration, spectrin, SNTF, calpain-2 inhibitors, therapeutics, neuropathology, neurodegeneration

## Abstract

Traumatic Brain Injury (TBI) is a major global health concern and a leading cause of death and disability, especially in young adults. It triggers complex secondary injury cascades, e.g., calcium dysregulation, mitochondrial dysfunction and protease activation, that extend well beyond the initial mechanical insult to drive ongoing neurodegeneration. The calcium-dependent protease calpain has emerged as a central mediator of TBI cellular pathology. Calpain cleaves a broad range of cytoskeletal and regulatory proteins across neuronal compartments, disrupting axonal integrity, synaptic function and calcium homeostasis. Despite decades of research, calpain remains an elusive therapeutic target. In this review, we examine the spatial and temporal patterns of calpain activation in the traumatically injured brain, categorize key calpain substrates by structure and location, and assess their mechanistic roles in TBI pathology. We also review recent advances in next-generation calpain-2 selective inhibitors with enhanced specificity and preclinical efficacy and discuss the emerging use of calpain-cleaved protein fragments such as SBDP145 and SNTF as candidate biomarkers for TBI diagnosis and progression. Drawing on molecular, preclinical, and clinical data, we argue that calpain warrants renewed attention as both a therapeutic target and mechanistic biomarker in TBI. It may be time for Cinderella to leave the basement.

## 1. Introduction

Traumatic Brain Injury, (TBI) is a preventable yet leading cause of morbidity and mortality that affects over 60 million individuals each year resulting in an estimated $US400 billion global economic burden [1,2]. Despite its high prevalence, TBI is a stubbornly difficult condition to treat because of marked heterogeneity in its clinical presentation, diverse injury mechanisms, and variable outcomes. There is no unified classification scheme for TBI which further complicates patient stratification and selection in clinical trial design, and likely contributes to the fact that no TBI clinical trial has demonstrated therapeutic efficacy to date [3,4]. TBI includes a primary mechanical insult followed by a secondary cascade of excitotoxicity, mitochondrial dysfunction, inflammation, loss of calcium homeostasis, and calpain activation [5,6]. This cascade results in neuronal and glial cell degeneration, cerebral edema, and neuroinflammation which in turn creates potential windows for therapeutic intervention [7].

**Figure 1:**
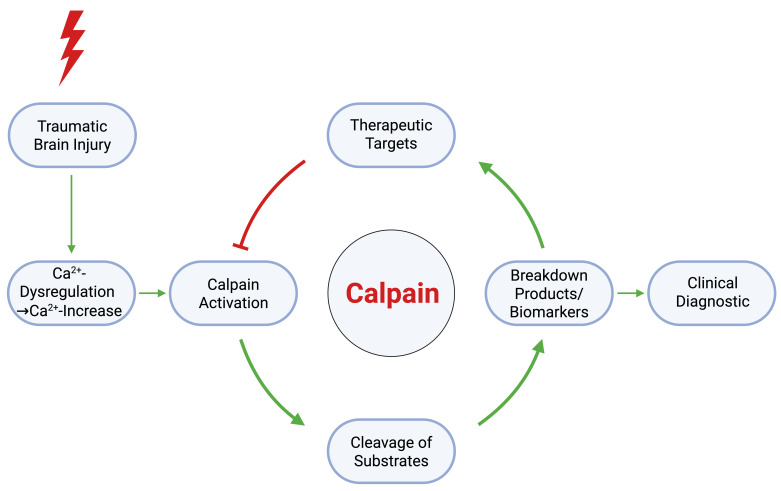
Schematic overview of calpain’s role in TBI pathophysiology and its translational applications. TBI disrupts calcium homeostasis, triggering calpain activation. Calpain-specific cleavage products serve as candidate biomarkers for clinical diagnosis and disease monitoring. Red arrows illustrate inhibition, green arrows indicate the sequence from injury to molecular disruption and potential intervention. This integrative model highlights calpain’s central role as both a pathogenic effector and a potential target for therapeutic and diagnostic development. Image created with BioRender.com.

Among the key mediators of secondary injury after TBI, calcium dysregulation and calpain activation have emerged as central drivers of progressive neuronal injury (Figure 1). Calpains are calcium-dependent cysteine proteases that cleave cytoskeletal, membrane-associated, and signaling proteins which promotes axonal degeneration and impaired neuronal function [8,9,10]. Sustained calpain activation is also implicated in chronic neurodegenerative disorders such as Alzheimer’s disease and related dementias, suggesting a potential mechanistic link to trauma-related neurodegeneration [11,12]. Although early TBI studies targeted calpains, enthusiasm declined after poor translational outcomes [13]. However, recent advances in selective calpain-2 specific inhibitors and improved understanding of TBI mechanisms have renewed interest in this pathway [13,14,15]. These developments support revisiting calpain inhibition as a therapeutic strategy in TBI.

## 2. Calpain Biology and TBI

### 2.1. Calpain and Regulation

Calpains are non-lysosomal calcium-dependent cysteine proteases first identified by Guroff in 1964 [16,17]. The name reflects their calcium-dependence (“cal”) and structural similarity to papain (“pain”) [18]. Humans express 15 calpain genes and 2 small regulatory subunit genes (Table 1). Catalytic isoforms are grouped into classical (e.g., calpain-1 and calpain-2) and non-classical types based on domain structure [19]. Calpain-1, (µ-calpain), and -2, (m-calpain), are the best characterized isoforms. Both are heterodimers composed of an 80 kDa catalytic subunit and a common 28 kDa regulatory subunit [17,20]. The large subunits share 55–65% structural homology, whereas the small regulatory subunit is identical in both isoforms [17]. The large subunit comprises four domains (I–IV), each with distinct functions [21]. Calcium binding to domain I triggers autolysis [22], while domain II contains the catalytic site responsible for substrate binding and protease activity [23]. Domain III, a C2-like domain, may mediate binding to calcium and phospholipids and serve a regulatory function [24]. Domain IV contains EF-hand motifs critical for calcium binding [25]. Calpain-1 and calpain-2 differ in calcium sensitivity and function: calpain-1, activated by micromolar calcium concentrations, is involved in synaptic plasticity [26,27] and supports processes like long-term potentiation (LTP) [28,29] whereas calpain-2, which is activated by millimolar calcium concentrations, limits LTP and is associated with neurodegeneration [28,30,31]. Both isoforms are abundant in the brain where their activity is tightly regulated by calpastatin, an endogenous inhibitor, which is thought to be calpain protease specific as well as responsive to intracellular calcium levels [17,18]. Calcium dysregulation after TBI disrupts this control, leading to aberrant calpain activation and neurological damage.

### 2.2. Loss of Calcium Homeostasis Following TBI

**Figure 2:**
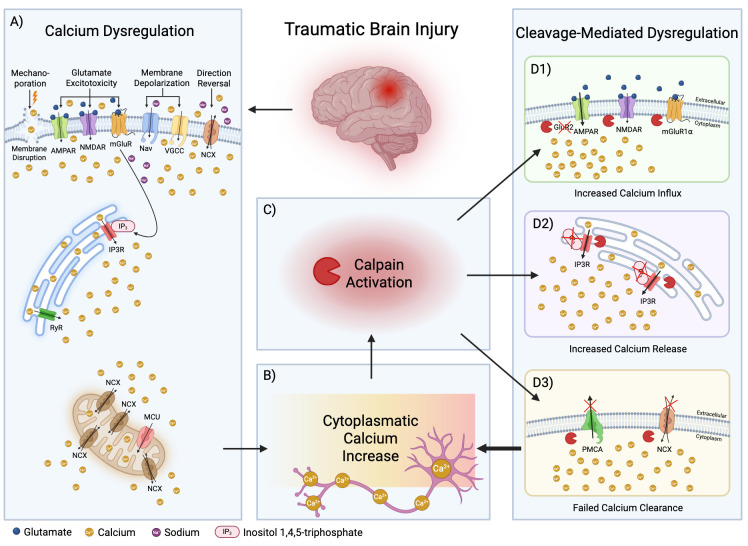
The Calpain-Ca^2+^-Loop is a self-perpetuating cycle triggered by TBI-induced calcium dysregulation. TBI initiates calcium influx through mechanoporation, glutamate excitotoxicity, membrane depolarization and reversal of calcium efflux pumps (**A**). Intracellular calcium increases rapidly (**B**) which leads to calpain activation (**C**). Calpain cleaves key regulatory proteins in the plasmalemma and ER including plasma membrane receptors, ER IP3 receptors, and calcium pumps, resulting in loss of calcium homeostasis, (**D1**–**D3**). This leads to sustained intracellular calcium elevation which drives further calpain activation, creating a feedforward cycle of progressive cellular damage. Image created with BioRender.com.

Physiological control of calcium homeostasis is essential for normal neuronal function, including synaptic plasticity and neurotransmission [6]. After TBI, intracellular calcium rises rapidly due to extracellular influx and intracellular release [6] (Figure 2(A,B)). One proposed mechanism is mechanoporation due to physical membrane disruption caused by compression, shearing, and stretching [67,68]. However, some evidence suggests that this may not account adequately for all calcium entry, pointing to other potential mechanisms [69]. A likely major contributor is glutamate excitotoxicity, whereby excess glutamate released from injured neurons activates ionotropic receptors, such as AMPA- (AMPAR’s) and NMDA- receptors (NMDAR’s), leading to calcium influx [70]. NMDAR’s are highly permeable to calcium [71], whereas AMPAR’s become calcium-permeable when the GluA2 subunit (formerly known as GluR2) is cleaved, as it can be observed under pathological calpain activation [72,73,74] (Figure 2(D1)).

Membrane depolarization after TBI activates voltage-gated sodium channels (Nav’s), and subsequently voltage-gated calcium channels (VGCC’s) [69,75,76,77], and reverses the direction of the sodium-calcium exchanger (NCX) [78,79,80,81], driving even more calcium into the cell.

Following TBI, intracellular calcium release also occurs from endoplasmic reticulum (ER) stores. Besides ionotropic receptors, glutamate can activate metabotropic receptors (mGluR’s) [82,83,84], leading to the production of the second messenger inositol 1,4,5-trisphosphate (IP3), which causes calcium release from the ER into the cytoplasm [84,85]. The process is amplified by calcium induced calcium release (CICR) from the ER through ryanodine receptors (RyR’s) [86,87]. Calpain-mediated cleavage of ER calcium channels induces ER dysfunction and passive calcium leak [88] (Figure 2(D2)).

Mitochondria, which normally buffer calcium through pumps and channels such as the mitochondrial calcium uniporter (MCU) [89], become overwhelmed with the sudden and massive influx after TBI, resulting in membrane depolarization, generation of reactive oxygen species (ROS), and energy failure [90,91,92]. Increased expression of the mitochondrial sodium-calcium exchanger (mNCX) after TBI suggests a compensatory response, but at the cost of reduced mitochondrial calcium buffering capacity [93].

Conflicting studies highlight that the calcium increase may originate from different sources depending on the underlying mechanism of injury. Some injury models are associated primarily with release of calcium from intracellular stores [94], whereas others show extracellular influx [69]. These discrepancies highlight the complexity of calcium dysregulation after TBI.

## 3. Calpain Substrates in the Traumatically Injured Brain

### 3.1. Cytoskeletal Substrates

**Figure 3:**
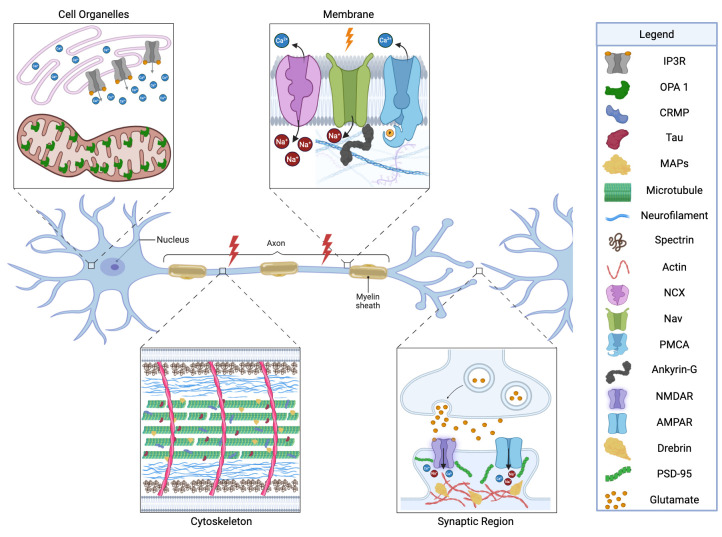
Subcellular localization of calpain substrates in neurons. Calpain targets are distributed across four major compartments: the cytoskeleton, membrane, synaptic region, and intracellular organelles. Proteolysis in each domain drives distinct pathological outcomes. Cytoskeletal cleavage disrupts axonal transport and structural integrity; membrane-associated substrate cleavage impairs ion channel stability and anchoring; synaptic protein degradation compromises neurotransmission and plasticity; and cleavage of organelle-associated targets perturbs calcium homeostasis and mitochondrial function. Lightning bolts denote sites of axonal injury. Image created with BioRender.com.

Neurons are the fundamental signaling units of the nervous system, characterized by a highly polarized morphology that relies on a dynamic cytoskeletal framework composed of microtubules, actin filaments, and neurofilaments [95,96,97]. Many of these cytoskeletal elements are calpain targets (Figure 3).

#### 3.1.1. Spectrin and ⍺II-Spectrin

In axons, dendritic spines, and presynaptic terminals, actin forms a periodic lattice with αII-spectrin and βII-spectrin, that is stabilized by ankyrin G [95,98]. In dendritic spines, branched actin networks create a core scaffold that supports synapse morphology and facilitates receptor trafficking [99], whereas in axon shafts, actin filaments are less abundant and exist in periodic circular actin rings that provide additional mechanical support to maintain axonal diameter [100].

One of the best characterized calpain targets is αII-spectrin. Post-traumatic cleavage by calpain generates two spectrin breakdown products (SBDP’s): SBDP145 (which is calpain-specific) and SBDP150 (which is also generated by caspase-3) [101,102,103]. SBDP145 is a reliable biomarker of calpain-specific proteolysis and is commonly used in preclinical studies to detect calpain activity post-TBI, because it is consistently elevated across multiple brain regions after injury [101,103,104,105,106,107,108,109,110,111,112]. Caspase-3 generates a distinct 120 kDa spectrin breakdown product, (SBDP120) [102]; however, SBDP120 shows no significant [103,113] or minimal [106,109,110] increase in brain tissue or CSF [111,112] after TBI, suggesting that calpain, rather than caspase-3, plays a more prominent role in cytoskeletal degradation after TBI.

A smaller calpain-derived spectrin fragment, the 17 kDa spectrin N-terminal fragment (SNTF), has been detected in injured gray and white matter after TBI in sheep [114]. SNTF has emerged as a promising candidate biomarker for both mild TBI (mTBI) [115] and long-term cognitive deficits [115]. In human studies, serum and plasma SNTF levels increased within 1 h of mTBI and remained significantly elevated from 12 h to 6 days after injury [116,117]. At 24 h, mean serum levels were 92% higher in TBI patients than healthy controls, with some individuals showing a 9-fold increase [115]. Elevated SNTF was significantly associated with persistent cognitive impairment [115]. Although sensitivity is limited, (~40%), its high specificity (approaching 100%), suggests that SNTF may be a useful adjunct biomarker for identifying patients at risk for chronic post-traumatic cognitive dysfunction [115], limiting its utility as a screening tool, but underscoring its utility as a potential prognostic biomarker.

Although αII-spectrin has been well-characterized, the degradation of βII-spectrin remains understudied. In a rat model of controlled cortical impact, βII-spectrin breakdown products (βsBDPs) were rapidly detected [118], exhibiting a similar temporal profile to αII-spectrin cleavage. However, βII-spectrin remains understudied with few in vivo models examining its role in TBI.

#### 3.1.2. Spatiotemporal Pattern of ⍺II-Spectrin Cleavage

**Figure 4:**
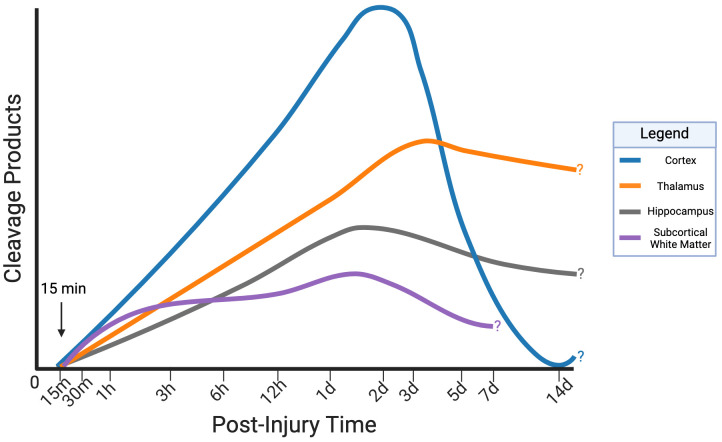
Proposed spatiotemporal model of SBDP145 in four different brain regions: cortex, thalamus, hippocampus, and subcortical white matter. Preclinical TBI models vary in methodology, limiting cross-study comparability. This schematic summarizes reported regional and temporal patterns in SBDP145 detection. Cortical accumulation is detectable by 15 min post-injury, with a distinct peak between 1–3 days. Thalamus, hippocampus and subcortical white matter exhibit slower but more sustained elevations. Arrow denotes the earliest reported detection. Question marks indicate timepoints beyond which data are not available. Chronic persistence remains largely uncharacterized. The time-axis is plotted logarithmically. Image created with BioRender.com.

Preclinical TBI studies using diverse animal models including CCI [103,107,108,111,113], impact acceleration injury (I/A) [105], closed head injury (CHI) [109], repeated blast overpressure (BOP) [110] and lateral [101], central [106], and medial fluid percussion injury (FPI) [104] report variable timing and distribution of spectrin cleavage products across different brain regions (Figure 4).

In the cortex, SBDP’s typically peak at 1–3 days, although some studies report increases as early as 15 min [107], others saw no changes until much later [101,103,104,106,108,109,110,111,113]. Clearance of SBDP’s is also variable with some studies reporting a return to baseline by day 7 [101,103,104,113] whereas others note sustained or delayed biphasic elevations until 14 days post-injury [106,108,109,110].

In contrast, the hippocampus and thalamus show slower but prolonged elevation of SBDP’s, sometimes persisting up to day 14 [101,104,108,109,113]. Whether one region is more affected than others remains controversial [101,104,108]. White matter regions, (e.g., corpus callosum, cingulum, and pyramidal tracts) tend to exhibit early but lower levels of SBDP’s compared to the gray matter, although the timepoint of detection varies across studies with some studies reporting increases as early as 15 min post-injury [101,104,105,106] and others finding no detectable elevation until 24 h [107].

Overall, inconsistencies in study design, timepoints, and outcome measures limit cross-study comparisons. Further, age-dependent vulnerability of the developing brain to injury and its impact on SBPD expression has not been examined. A more standardized approach to TBI investigation is needed to help fully map the spatiotemporal dynamics of calpain activity during injury progression.

#### 3.1.3. Neurofilaments

Neurofilaments (Nf) are a class of intermediate filaments, highly enriched in myelinated axons, where they regulate axon diameter by spacing microtubules [96,119].

All three neurofilament subunits (Nf-L, Nf-M, and Nf-H) are calpain substrates, with cleavage observed in both in vitro and in vivo models when calcium is elevated [120,121,122,123]. However, very low-level calpain-mediated cleavage of Nf also occurs under physiological conditions, suggesting that calpain may contribute to both pathological and physiological Nf turnover [122]. Excitotoxicity increases calpain-mediated cleavage and extracellular release of Nf fragments, particularly Nf-L [121]. Although two calpain cleavage sites have been mapped for bovine Nf-M, species differences limit translatability to humans [120]. The structural determinants of Nf subunit specific susceptibility for calpain cleavage have not yet been fully characterized [120].

#### 3.1.4. Microtubules and Microtubule-Associated Proteins (MAP’s)

Microtubules composed of α- and β-tubulin dimers are essential for intracellular trafficking [97]. In axons, they are uniformly oriented for efficient anterograde and retrograde transport via kinesin and dynein motor proteins, whereas in the soma and dendrites they show less uniform arrangement [124,125,126]. Microtubule-associated proteins (MAPs) including tau (axonal), MAP2 (dendritic), and MAP1 (both), promote microtubule assembly, and help to stabilize and organize these structures [127,128,129]. Calpain cleaves both microtubules and MAPs, disrupting cytoskeletal integrity after TBI [130,131,132]. MAP1 and MAP2 are highly sensitive to calpain-mediated degradation, with rapid loss following calpain activation [131,132]. Tubulin is more resistant and is only significantly cleaved at higher enzyme concentrations or under strong activating conditions [131,133,134].

Tau is a prominent microtubule associated protein target with rapid C-terminal digestion by calpain within minutes of exposure [135,136,137]. Calpain-mediated cleavage of tau, tubulin, and associated proteins compromises microtubule integrity, leading to a loss of structural integrity and impaired axonal transport.

#### 3.1.5. Collapsin Response Mediator Proteins (CRMP’s)

CRMP’s (CRMP 1-5) interact with microtubules and actin to regulate axon guidance, dendritic branching, and synaptic plasticity [138]. Among these, CRMP-1, CRMP-2, CRMP-3 and CRMP-4 are known calpain substrates after TBI, whereas CRMP-5 is resistant to cleavage [139,140]. Following CCI in rats, calpain-2-mediated cleavage generates CRMP-2 (55 kDa) and CRMP-4 (58 kDa) breakdown products in the cortex and hippocampus, implicating a role in gray matter remodeling [139]. In vitro excitotoxicity studies have also demonstrated calpain-mediated cleavage of CRMP-1 and CRMP-3, followed by nuclear translocation [140,141].

### 3.2. Calpain Targets of the Membrane/Axolemma

The axolemma (specialized plasma membrane of the axon), plays an essential role in signal conduction and homeostasis. In myelinated axons, voltage gated potassium channels (e.g., Kv1.1, Kv1.2, Kv7.2, Kv7.3) and sodium ion channels (Nav1.1, Nav1.2, and Nav1.6) localize to the axon initial segment (AIS) and nodes of Ranvier (NOR), for saltatory conduction [142,143], whereas unmyelinated axons have ion channels and pumps dispersed along the entire length of the membrane [144]. Calcium homeostasis is facilitated by sodium-calcium exchangers (NCX) and plasma membrane calcium ATPase (PMCA), as well as scaffolding proteins like ankyrin-G [98] which help to organize channels [145].

#### 3.2.1. Sodium Channels and Ankyrin-G

Voltage-gated sodium channels (Nav) such as Nav1.2 and Nav1.6 are rapidly cleaved by calpain after TBI [146,147,148,149]. In a swine concussion model, Nav1.6 was reduced at the white matter nucleolar organizer region (NOR) within 6 h post-injury, a pattern similarly observed in human autopsy brain [146]. Calpain preferentially targets intracellular loops of the α-subunit [148], particularly the III-IV linker resulting in persistent Na^+^ influx, which amplifies pathological accumulation of calcium in a feed-forward cycle promoting further proteolysis and axonal degeneration [147].

Calpain also cleaves the anchoring molecule, ankyrin-G [106]. In a rat lateral fluid percussion TBI model, ankyrin-G breakdown products accumulated at 3 h post-injury in the corpus callosum but were limited in the cortex suggesting region specific activation effects [106]. Cleavage of ankyrin-G by calpain disrupts cytoskeletal integrity and axonal conduction [98,106].

#### 3.2.2. Failed Calcium Clearance Mechanisms

Calpain selectively cleaves the sodium-calcium exchanger 3 (NCX3), the primary NCX isoform implicated in excitotoxic neuronal injury (Figure 2(D3)). This has been repeatedly demonstrated in cerebellar granule neurons and hippocampal neurons following overactivation of glutamate receptors (NMDA or AMPA subtypes) or brain ischemia [150,151,152], whereas NCX1 and NCX2 tend to be spared [150,152]. Cleavage of NCX3 results in specific breakdown fragments [150] and leads to impaired calcium extrusion capacity, causing neuronal calcium overload and increased neuronal injury and death [150,152]. Similarly, calpain activation disrupts the plasma membrane calcium ATPase (PMCA) function (Figure 2(D3)) [153]. Exposure to high concentrations of glutamate (200 μM) double the time constant from ~8.8 to ~16.5 s, reducing PMCA-mediated calcium efflux and leading to further calcium overload [153].

### 3.3. Synaptic Targets

The synapse is a specialized compartment that promotes intercellular communication between neurons [154,155]. The presynaptic terminal contains neurotransmitter-filled vesicles and active zones for release which is supported by a presynaptic scaffold for vesicle trafficking and is rich in voltage- and calcium-gated channels [156,157,158,159,160]. The postsynaptic density (PSD), which is typically found on dendritic spines, includes AMPA and NMDA-type glutamate receptors, scaffolding proteins (e.g., PSD-95, GKAP, Shank, and Homer) and signaling molecules that help to organize and coordinate synaptic signals [155].

#### 3.3.1. Postsynaptic Scaffolds

Several postsynaptic protein components (e.g., PSD-95, drebrin, AMPA- and NMDA-receptors) are calpain substrates [26,34,161,162,163,164,165,166,167,168]. PSD-95, a central scaffold protein anchoring NMDA and AMPA receptors is cleaved by calpain into 50 kDa and 36 kDa fragments [162], disrupting postsynaptic density architecture [34]. Although important for physiologic responses, this process is amplified and more pronounced during excitotoxic conditions such as TBI-related NMDA receptor activation or brain ischemia [34,162,163]. However, findings have varied regarding the extend and completeness of PSD-95 cleavage. Calpain treatment of synaptic membranes in vitro results in only about 50% maximal degradation of PSD-95, regardless of increasing calpain concentration [162]. In contrast, using purified PSD fractions, it is possible to observe almost complete degradation (near 100%) of PSD-95 [34]. The more purified PSD preparations may facilitate better calpain access to PSD-95, and thereby more complete proteolysis. In less purified synaptic membrane fractions, PSD-95 may be physically shielded or engaged in stable complexes, making it less accessible to calpain.

Drebrin is an evolutionarily conserved F-actin-binding protein localized to dendritic spines that is crucial for synaptic structure and function [95,169]. Both in vitro and in vivo, drebrin is degraded by calpain after calcium influx triggered by overactivation of NMDA-receptors [164]. However, the role of drebrin in TBI pathology remains unclear.

#### 3.3.2. Glutamate Receptors

Calpain cleaves the C-terminal domain of AMPA receptor subunits (Figure 2(D1)), especially GluR1 and GluR2, and possibly GluR3, modulating synaptic plasticity under physiological conditions and contributing to cellular dysfunction in pathological states [161,166]. AMPA receptors lacking the GluR2 subunit, become highly permeable to calcium, increasing vulnerability to calpain mediated proteolysis after TBI [72,73,74].

NMDA receptors also function as calpain targets (Figure 2(D1)). The C-terminal domains of the NR2A and NR2B subunits are preferentially cleaved, at the same time the NR1 subunit is more resistant to cytological stress [26,165,167,168]. Although truncated receptors retain basic functions, cleavage disrupts interactions with scaffolding and signaling proteins, contributing to the development of cellular pathology [26].

Calpain also cleaves mGluR1⍺, a member of group I mGluR’s, (Figure 2(D1)) [170]. Truncation of mGluR1⍺ in the C-terminus yields 100 kDa and 38 kDa fragments [170]. Despite cleavage, the truncated receptor is functionally active and retains the ability to increase cytosolic calcium [170].

### 3.4. Organelle Targets

Calpains affect organelle-associated proteins to disrupt cellular physiology beyond the cytoskeleton, membrane, and synapse following TBI.

#### 3.4.1. Endoplasmic Reticulum

The inositol 1,4,5-trisphosphate receptor (IP3R) located on the endoplasmic reticulum membrane plays a major role in regulating calcium homeostasis [86]. Incubation of membrane fractions or purified IP3R with calcium and cerebellar cytosol, leads to receptor degradation and the generation of ~130 kDa and ~95 kDa fragments, likely from the C-terminus of the protein [171]. Calpain-mediated cleavage of IP3R produces dysregulated fragments with InsP3-independent activity, thereby increasing ER calcium leak and further sensitizing neurons to excitotoxic damage (Figure 2(D2)) [88]. Calpain-mediated IP3R cleavage not only degrades the receptor but also creates leaky channels driving increased cytoplasmic calcium, to create a feedforward cycle of calpain activation and neuronal injury following TBI.

#### 3.4.2. Mitochondria

A major mitochondrial calpain substrate is optic atrophy 1 protein (Opa1), a GTPase located on the mitochondrial inner membrane, which is essential for mitochondrial fusion and cristae structure [172]. Calpain cleavage of Opa1 results in mitochondrial fragmentation and cristae dilation [172,173], whereas calpain inhibition preserves Opa1 function and mitochondrial integrity [172].

### 3.5. Emerging Targets

The Protein Tyrosine Phosphatase, Non-Receptor Type 13 (PTPN13) is a brain enriched phosphatase and a selective substrate for calpain-2, but not calpain-1 [174]. Calpain-2 cleavage inactivates PTPN13, producing stable breakdown products (P13BPs) and leading to disinhibition of the non-receptor tyrosine kinase c-Abl, which results in increased c-Abl activity [174]. P13BP breakdown products are detectable in the blood after TBI, and positively correlate with injury severity, [175]. P13BP levels rise acutely after TBI, peak around 24 h, and normalize at around 10 days [175]. However, the etiological specificity of P13BP for TBI versus other neurologic disorders, (e.g., stroke, epilepsy, neuroinflammation) remains to be determined.

## 4. Calpain in the Transition from Acute TBI to Chronic Neurodegeneration and SARM1

As discussed above, calpain is a key effector molecule in the pathology of acute TBI, driving early pathological cascades through rapid and widespread proteolysis. However, calpain’s activity may extend well beyond the initial injury. Following CCI in rats and repeat blast exposures in mice, sustained calpain activity occurs in multiple brain regions for up to 14 days post-injury (Figure 3) [108,110], and includes a second delayed peak in calpain-specific breakdown products [110]. This observation indicates that calpain is not only a central player in acute brain injury but also as a potential driver of progressive neurodegeneration after TBI.

This hypothesis is supported by calpain’s established role in several neurodegenerative diseases, including chronic traumatic encephalopathy (CTE) [176], Alzheimer’s disease (AD) [177,178,179,180], Parkinson’s disease (PD) [181], Amyotrophic lateral sclerosis (ALS) [182], Huntington’s disease (HD) [183], and Multiple Sclerosis (MS) [184,185]. Calpain-mediated cleavage can generate misfolded and aggregation prone protein fragments. An example is the protein tau which is cleaved by calpain after TBI [135,136,137]. Once cleaved by calpain, tau may become more prone to hyperphosphorylation and aggregation, leading to pathological tauopathy, a hallmark of Alzheimer’s disease and CTE [178].

A key link between acute TBI and long-term neurodegeneration is white matter axonal loss, a process that involves both calpain activation and the sterile alpha TIR motif-containing protein 1 (SARM1). SARM1 is a central mediator of axonal degeneration, and after TBI is activated and rapidly depletes nicotinamide adenine dinucleotide (NAD+) [186], causing a metabolic energy crisis and axonal fragmentation [187]. This process underlies Wallerian degeneration of white matter, a common finding across many chronic neurodegenerative diseases [188]. Notably, SARM1 knockout confers strong neuroprotection after TBI, preserving axonal structure and function [189].

Given their shared roles in axonal injury and neurodegeneration, potential interactions between calpain and SARM1 have drawn increasing interest. Although both proteins have been extensively studied in isolation, their relationship remains undefined, and it is unclear whether or how they influence each other. Recent work in hippocampal neurons suggests potential compartment-specific roles for SARM1 and calpain [190]. In dendrites, calpain-2 is activated downstream of SARM1 via calcium influx whereas in axons, SARM1-mediated axonal degeneration has been reported to be calpain independent, raising questions about potential differences in calpain isoform distribution across neuronal subtypes (e.g., excitatory versus inhibitory) or even across cellular compartments [190]. For example, in hippocampal neurons, calpain-2, the dominant isoform associated with neuronal pathology [31], is preferentially enriched in dendrites, which could potentially account for the lack of parallel activation observed in axons despite a similar loss of calcium homeostasis across the cell.

## 5. Translational Aspects of Calpain Biology

### 5.1. Biomarkers

Currently, diagnoses of TBI tend to be somewhat subjective and rely primarily on neuroimaging, neurological scoring systems, and clinical signs and symptoms, which may be obscured in unconscious, sedated, or intoxicated patients [191,192,193]. Biomarkers have recently emerged as a complementary clinical adjunct for improved diagnostic accuracy, guiding treatment, and predicting outcomes [3,194]. Currently, the blood-based biomarkers glial fibrillary acidic protein (GFAP) and ubiquitin C-terminal hydrolase-L1 (UCH-L1) are approved by the US Food and Drug Administration (FDA) for evaluating suspected mTBI [195,196,197] (Table 2). Both exhibit high sensitivity and negative predictive values, allowing effective exclusion of acute intracranial pathology and reducing reliance on CT scans, thereby lowering costs and radiation exposure [198]. However, unlike GFAP and UCH-L1, calpain-generated fragments such as SBDP145 and SNTF have not yet undergone full analytical or clinical validation. Although these markers are promising candidates, their translation into clinical diagnostic assays will require rigorous confirmation of individual performance characteristics, standardization across testing platforms and the biofluids to be tested, characterization of sensitivity, specificity and predicative values, and establishing laboratory ranges and diagnostic cutoffs in accordance with regulatory standards.

Although not yet FDA approved as clinical biomarkers, several calpain-specific breakdown products have recently emerged as potential candidate biomarkers in cerebrospinal fluid (CSF) and blood. After TBI, the cleavage products of αII-Spectrin—SBDP’s and SNTF—rise rapidly. SBDP’s are primarily detected in the CSF [111,112] whereas SNTF is more readily measured in the blood, (see Section 3.1.1.) [115]. These calpain-generated cleavage products may offer specific insights into axonal pathology in TBI, given αII-Spectrin’s axonal location compared to somatic (NSE, UCHL-1) or astrocytic (S100B, GFAP) markers [10]. However, translating these findings from animal models to humans remains challenging. For example, calpain-specific SBDP145 peaks at 72 h in rat CSF [111], but peaks as early as 6 h after TBI in humans [112]. Although SBDP’s and SNTF are not yet used in routine clinical diagnostics, they have proven valuable as preclinical tools for investigating axonal pathology after TBI [10].

### 5.2. Previous and Current Approaches of Calpain-Inhibitors

Despite the public health burden imposed by TBI there are currently no approved pharmacological treatments to reduce or prevent long-term neurological damage [199,200]. Calpain has long been a therapeutic target of interest because of its broad substrate range and central role in TBI pathophysiology.

Early efforts using non-selective calpain-inhibitors, such as SNJ-1945 and MDL-28170, did not show efficacy in TBI models [201,202]. However, these compounds inhibited both calpain-1 and calpain-2, despite their diverging roles. In general, calpain-1 is thought to be neuroprotective, whereas calpain-2 is primarily associated with neurodegeneration and TBI pathophysiology [31]. Non-selective inhibition likely negated the potential neuroprotective effects of calpain-1. In response, recent strategies have focused on selective calpain-2 inhibitors [14,30,31]. These compounds aim to block the pathological effects of calpain-2 activation while preserving calpain-1’s potentially beneficial neuroprotective function [30,31].

NA-101 was one of the first selective calpain-2 inhibitors studied. In mice who underwent CCI, NA-101 significantly reduced calpain-2 activity and led to decreased lesion volume and improved motor and cognitive outcomes [31]. However, higher doses caused neurotoxicity with poor inhibition of calpain-2 at the lesion margins, suggesting limited drug penetration into severely damaged tissue [31]. These limitations led to the development of a more potent analogue, NA-184 [30]. In preclinical animal studies, NA-184 showed dose-dependent inhibition of TBI-induced calpain-2 activity without affecting calpain-1[30], crossed the blood-brain barrier effectively, and reduced neuronal death, lesion volume, and behavioral deficits post-TBI [30]. Although selective calpain-2 inhibitors have demonstrated efficacy in preclinical studies, they have not yet advanced to human clinical trials.

Both, non-selective and selective calpain-inhibitors target the catalytic domain (domain II) of calpain, binding covalently and reversibly to the active site. MDL-28170 contains a dipeptidyl aldehyde warhead, whereas SNJ-1945, NA-101 and NA-184 use alpha-ketoamides. Compared to other warhead types, alpha-ketoamides show superior potency, enhanced permeability, and more favorable binding interactions [203,204].

Isoform selectivity is achieved by exploiting subtle structural differences between calpain 1 and 2 rather than using distinct inhibitory mechanisms. For example; differences in domain III modulate the binding pocket at the catalytic site; resulting in the formation of a key hydrogen bond between the inhibitor and calpain-2; but not calpain-1 [14]. Further optimization of isoform substituents guided by isoform-specific structural features of calpain-1 and calpain-2; may further improve inhibitor selectivity and stability [14].

## 6. Conclusions

Calpain, once an overlooked player in TBI research, has recently reemerged as a central protein linking acute TBI to long-term trauma-related neurodegeneration. The recognition that isoform specific forms, especially calpain-2, can drive the deleterious pathology of TBI has opened new therapeutic options with selective inhibitors such as NA-184 already showing promise in pre-clinical settings. Further, calpain-derived breakdown products show promise as novel diagnostic, predictive and prognostic biomarkers in TBI. When integrated with existing tools these candidate biomarkers may enhance clinical decision making in a new era of precision-guided TBI care. Yet much remains unknown, including the potential interactions between calpain, SARM1, cellular aging, and chronic neurodegeneration after TBI. Like Cinderella arriving at the ball, calpain is now emerging in a new light and is ready to be seen for its full potential. Translating the neuroprotective effects of targeting calpain into better functional outcomes after TBI will require: (i) advancing calpain-selective inhibitors into clinical trials; (ii) defining its distinct and overlapping effects in acute and chronic injury, and (iii) integrating calpain-based biomarkers into routine clinical use.

## Figures and Tables

**Table 1:** Overview of Calpain Isoforms and Small Regulatory Subunits.

Name	Gene	Tissue Distribution	Physiological Function	Associated Pathology	Refs.
Calpain-1 *	CAPN1	Ubiquitous; high in brain, muscle	Synaptic plasticity, learning (LTP), axon growth	Hereditary spastic paraplegia; Spinocerebellar ataxia	[26,27,29,31,32,33]
Calpain-2 *	CAPN2	Ubiquitous; high in brain, heart	Cytoskeletal regulation, cell migration, embryogenesis, cell proliferation	Neurodegenerative diseases; Cardiomyopathy	[34,35,36,37,38,39]
Calpain-3 *	CAPN3	Skeletal muscle	Muscle repair and maintenance, Sarcomere remodeling	Limb-girdle muscular dystrophy type 2A (LGMD2A)	[40,41,42]
Calpain-5	CAPN5	Ubiquitous; high in retina, brain	Signal transduction	Autosomal dominant neovascular inflammatory vitreoretinopathy (ADNIV)	[43,44]
Calpain-6	CAPN6	Placenta; embryonic muscle	No proteolytic activity, involved in cytoskeletal dynamics	Cancer progression	[45,46,47,48]
Calpain-7	CAPN7	Ubiquitous	Cell division, endosomal trafficking/receptor turnover	Hepatocellular carcinoma (HCC)	[49,50,51]
Calpain-8 *	CAPN8	Gastro- intestinal; stomach	Cell migration and proliferation, maintenance of gastric mucosal integrity	Inflammatory gastric diseases	[52]
Calpain-9 *	CAPN9	Gastro- intestinal	Cell migration and proliferation, maintenance of gastric mucosal integrity	Inflammatory gastric diseases	[52]
Calpain-10	CAPN10	Ubiquitous; high in pancreas, brain	Cytoskeletal remodeling, insulin secretion in pancreatic β-cells	Type 2 diabetes mellitus (T2DM)	[53,54,55]
Calpain-11 *	CAPN11	Testis	Cytoskeletal remodeling, spermatogenesis, meiosis and sperm functional processes	Unknown	[56]
Calpain-12 *	CAPN12	Skin; hair follicles	Epidermal ontogenesis, hair follicle cycling	Congenital ichthyosis	[57,58]
Calpain-13 *	CAPN13	Ubiquitous	Unknown	Cerebral ischemia- reperfusion injury	[59]
Calpain-14 *	CAPN14	Esophagus	Unknown, potentially epithelial barrier regulation	Eosinophilic esophagitis (EoE)	[60]
Calpain-15	CAPN15	Ubiquitous	Unknown, potentially neurodevelopment	Congenital eye anomalies and other neurodevelopmental deficits	[61,62]
Calpain-16	CAPN16	Ubiquitous	Unknown, presumed to lack proteolytic function as it only encodes the N-terminal half of the catalytic domain	Unknown	[63]
Calpain-4/ Calpain Small Subunit 1	CAPN4/ CAPNS1	Ubiquitous	Regulatory subunit	Cancer progression	[20,64]
Calpain Small Subunit 2	CAPNS2	Ubiquitous	Unknown	Unknown	[65,66]

This table summarizes human calpain isoforms, including their assumed physiological roles and isoform-specific associated pathologies; Calpain Small Subunit 1 formerly carried the name calpain-4, but is solely regulatory, not catalytic; * indicates classical calpains; calpains without * indicate non-classical calpains; Refs.: references.

**Table 2:** Comparative Overview of Established and Emerging TBI Biomarkers.

Biomarkers	Sample Matrix	Detection Window	Calpain Specificity	Sensitivity/ Specificity	Application	Refs.
GFAP/ UCH-L1	Blood (serum)	Within 12h post-TBI	Not applicable	High/ moderate	FDA approved clinical biomarker for mTBI CT necessity assessment	[196,197,198]
SBDP145	CSF (primarily); blood (serum)	6h-7d post-TBI	High	Moderate/ not applicable	Preclinical/ clinical studies	[191]
SBDP150	CSF (primarily)	6h-5d post-TBI	Low (also generated by caspase-3)	Low- moderate/ not applicable	Preclinical/ clinical studies	[191]
SNTF	Blood (serum/ plasma)	1h-6d post-TBI	High	Low- moderate/ High	Preclinical/ clinical studies	[115,116,117]

Comparison of FDA-approved TBI biomarkers GFAP and UCH-L1 with calpain-derived spectrin breakdown products, (SBDP145, SBDP150, and SNTF). Detection windows for GFAP and UCH-L1 reflect current clinical diagnostic use. Detection windows for calpain breakdown products are based on reported durations in preclinical studies and the performance characteristics remain to be rigorously defined; h: hours; d: days; Refs.: references.

## Data Availability

Not applicable, no new data were created.

## Abbreviations

TBI	Traumatic Brain Injury
LTP	Long-Term Potentiation
Refs.	References
AMPA(-R)	α-Amino-3-Hydroxy-5-Methyl-4-Isoxazolepropionic Acid (-Receptor)
NMDA(-R)	N-Methyl D-Aspartate (-Receptor)
VGCC	Voltage-Gated Calcium Channel
NCX	Sodium-Calcium Exchanger
mGluR	Metabotropic Glutamate Receptor
IP3(-R)	Inositol 1,4,5-Trisphosphate (-Receptor)
CICR	Calcium Induced Calcium Release
ER	Endoplasmic Reticulum
RyR	Ryanodine Receptor
MCU	Mitochondrial Calcium Uniporter
ROS	Reactive Oxygen Species
mNCX	Mitochondrial Sodium-Calcium Exchanger
SBDP	Spectrin Breakdown Product
SNTF	Spectrin N-Terminal Fragment
βsBDP	β-Spectrin Breakdown Product
CCI	Controlled Cortical Impact
I/A	Impact Acceleration Injury
CHI	Closed Head Injury
BOP	Blast Overpressure
FPI	Fluid Percussion Injury
Nf(-L, -M, -H)	Neurofilament (-light, -medium, -heavy)
MAP	Microtubule-Associated Protein
CRMP	Collapsing Response Mediator Protein
Kv	Voltage- Gated Potassium Channel
Nav	Voltage-Gated Sodium Channel
AIS	Axon Initial Segment
NOR	Nodes of Ranvier
PMCA	Plasma Membrane Calcium ATPase
NOR	Nucleolar Organizer Region
PSD(-95)	Post-Synaptic Density(-95)
Opa1	Optic Atrophy 1 Protein
PTPN13	Protein Tyrosine Phosphatase, Non-Receptor Type 13
CTE	Chronic Traumatic Encephalopathy
AD	Alzheimer’s Disease
PD	Parkinson’s Disease
ALS	Amyotrophic Lateral Sclerosis
HD	Huntington’s Disease
MS	Multiple Sclerosis
SARM1	Sterile Alpha TIR Motif-Containing Protein 1
NAD+	Nicotinamid Adenine Dinucleotide
GFAP	Glial Fibrillary Acidic Protein
UCH-L1	Ubiquitin C-Terminal Hydrolase-L1
FDA	Federal Drug Administration
mTBI	Mild Traumatic Brain Injury
CSF	Cerebrospinal Fluid
NSE	Neuron-Specific Enolase
S100B	Calcium Binding Protein 100B
